# Effect of secondary infection on epithelialisation and total healing of cutaneous leishmaniasis lesions

**DOI:** 10.1590/0074-02760160557

**Published:** 2017-09

**Authors:** Liliane de Fátima Antonio, Marcelo Rosandiski Lyra, Maurício Naoto Saheki, Armando de Oliveira Schubach, Luciana de Freitas Campos Miranda, Maria de Fátima Madeira, Maria Cristina da Silva Lourenço, Aline Fagundes, Érica Aparecida dos Santos Ribeiro, Leonardo Barreto, Maria Inês Fernandes Pimentel

**Affiliations:** 1 Fundação Oswaldo Cruz-Fiocruz, Instituto Nacional de Infectologia Evandro Chagas, Laboratório de Pesquisa Clínica e Vigilância em Leishmanioses, Rio de Janeiro, RJ, Brasil; 2 Fundação Oswaldo Cruz-Fiocruz, Instituto Nacional de Infectologia Evandro Chagas, Laboratório de Bacteriologia, Rio de Janeiro, RJ, Brasil; 3 Conselho Nacional de Desenvolvimento Científico e Tecnológico, Rio de Janeiro, RJ, Brasil; 4 Fundação Carlos Chagas Filho de Amparo à Pesquisa do Estado do Rio de Janeiro, Rio de Janeiro, RJ, Brasil

**Keywords:** cutaneous leishmaniasis, wound infection, opportunistic infection

## Abstract

**BACKGROUND:**

Cutaneous leishmaniasis (CL) generally presents with a single or several localised cutaneous ulcers without involvement of mucous membranes. Ulcerated lesions are susceptible to secondary contamination that may slow the healing process.

**OBJECTIVE:**

This study verified the influence of non-parasitic wound infection on wound closure (epithelialisation) and total healing.

**METHODS:**

Twenty-five patients with a confirmed diagnosis of CL and ulcerated lesions underwent biopsy of ulcer borders. One direct microbial parameter (germ identification in cultures) and four indirect clinical parameters (secretion, pain, burning sensation, pruritus) were analysed. FINDINGS Biopsies of ten lesions showed secondary infection by one or two microorganisms (*Staphylococcus aureus*, *Pseudomonas aeruginosa*, *Enterococcus faecalis*, *Streptococcus pyogenes* and *Candida parapsilosis*). “Secretion” and “burning sensation” influenced epithelialisation time but not total healing time. Positive detection of germs in the ulcer border and “pain” and “pruritus” revealed no influence on wound closure.

**CONCLUSIONS:**

Our borderline proof of clinical CL ulcer infection inhibiting CL wound healing supports the need to follow antimicrobial stewardship in CL ulcer management, which was recently proposed for all chronic wounds.

Cutaneous leishmaniasis (CL) is endemic in the State of Rio de Janeiro (RJ), Brazil, with 548 confirmed cases between 2007 and 2015 (MS 2016). Transmission occurs via the bite of sand flies infected by parasites of the genus *Leishmania* (MS 2017).

*Leishmania (Viannia) braziliensis* is the primary causative species of CL in RJ (Marzochi & Marzochi 1994). CL typically presents with painless ulcerated skin lesions, generally as a single or several ulcers in skin areas exposed to insect bites that display a rounded or oval shape, infiltrated base, well delimited and raised erythaematous borders, reddish background with gross granulations and little secretion (MS 2017). The most commonly used treatment is meglumine antimoniate. The mean epithelialisation time of lesions after treatment is 30 to 60 days (Schubach et al. 2005).

The presence of bacterial secondary infection may cause local pain and produce serous and purulent exudate that completely or partially recovers the ulcer and subsequently dries into crusts (MS 2017), and it may interfere with the healing process (Navarro et al. 2009, Sadeghian et al. 2011).

The incidence of secondary infection in CL lesions ranges from 23.6-81% (Vera et al. 2001, Gonçalves et al. 2009, Sadeghian et al. 2011). The most frequently isolated bacteria from cutaneous lesions of leishmaniasis are *Staphylococcus aureus*, *Staphylococcus epidermidis*, *Pseudomonas aeruginosa*, *Klebsiella pneumoniae* and *Corynebacterium diphtheriae* (Vera et al. 2001, Gonçalves et al. 2009, Navarro et al. 2009, Sadeghian et al. 2011).

Navarro et al. (2009) recommended the use of an antibiotic associated with meglumine antimoniate for the treatment of CL in Venezuela. Sadeghian et al. (2011) observed a decreased therapeutic effect of meglumine antimoniate in cutaneous lesions with secondary bacterial infection. These authors suggested evaluations of CL lesions for bacterial infection prior to retreatment in cases of non-response to specific treatments. However, Vera et al. (2001) observed no influence of a secondary bacterial infection restricted to the ulcer in the complete re-epithelialisation process of CL lesions.

We verified whether the occurrence of secondary infection by bacteria or fungi in CL ulcers influenced the time for wound closure (i.e., epithelialisation) and total healing.

## MATERIALS AND METHODS

This report was a prospective cohort study of patients with a confirmed diagnosis of localised CL with ulcerated lesions who were treated at the Laboratory of Clinical Research and Surveillance in Leishmaniasis, Evandro Chagas National Institute of Infectious Diseases (INI), Oswaldo Cruz Foundation (Fiocruz), RJ, Brazil, between October 2013 and February 2016.

We excluded patients who presented with more than five skin lesions, who did not submit to biopsy at INI, who did not sign an informed consent form, or who were pregnant. The parasitological diagnosis of CL was established via direct smear examination and/or culture, polymerase chain reaction (PCR) or histopathological examination of lesion fragments with visualisation or isolation of the protozoan or its DNA.

We collected information on co-morbidities and systemic and/or topical antibiotic and/or antifungal therapies in the 30 days prior to the biopsy procedure during the diagnostic investigation phase. The largest or most infiltrated lesion was selected in patients with more than one cutaneous lesion. Biopsy procedures included asepsis of the lesion with a 70% ethyl alcohol solution, local anaesthesia with 2% lidocaine without a vasoconstrictor, and incision of the lesion border using a scalpel or “punch” (5 to 6 mm in diameter). The cutaneous sample was divided into six fragments, and each one was sent for the following tests: histopathological analysis; culture, PCR and immunohistochemistry for *Leishmania* (Fagundes et al. 2010, Almeida et al. 2011, Quintella et al. 2012); culture for bacteria; and culture for fungi.

Fragments for bacterial culture were placed in a sterile flask containing a 0.9% NaCl solution for transportation. Flasks were subsequently sent for growth and identification of aerobic and/or facultative anaerobic bacteria and mycobacteria. Fragments were macerated and inoculated into the following culture media: blood agar with 5% of Columbia Agar Base supplemented with defibrinated sheep blood (Merck, Darmstadt, Germany); chocolate agar supplemented with factor V and X (Merck, Darmstadt, Germany), agar brolacin (Merck, Darmstadt, Germany), and thioglycollate medium (Merck, Darmstadt, Germany). We identified bacterial species using conventional biochemical methods (Garcia & Isenberg 2007) and automated analyses in Vitek 2 Compact equipment (BioMerieux Inc., Durham, NC, USA) according to the manufacturer’s specifications. Culture for mycobacteria was performed in Lowenstein-Jensen medium and automated culture using the BACTECTM MGIT™ 960 Mycobacterial Detection System (BD - Becton Dickinson/Franklinlakes/Nova Jersey/EUA).

We interpreted sensitivity tests using the criteria in the M100-S24 document (CLSI 2013, 2014, 2015).

Fragments for fungi isolation were cultured on Sabouraud dextrose agar media (SDA) and Mycosel agar (Myc) at 25ºC for up to four weeks. We identified filamentous fungi based on morphology, and yeasts were identified using automated biochemical methods in Vitek 2 Compact equipment (BioMerieux Inc., Durham, NC, USA).

All patients were treated with meglumine antimoniate. Nine patients received successful subcutaneous therapy with this medication, which was administered as one to three injections with 15 to 20 days intervals according to the intralesional treatment protocol at our institution (Vasconcellos et al. 2010). Fifteen patients received intramuscular injections of 5 mg of antimony per kilogram per day for 30 days, which is the standard systemic treatment at our institution (Schubach et al. 2005), and one patient received intramuscular antimony (20 mg/kg/day) for 20 days (Brazilian Ministry of Health recommendation for treatment of CL patients). Attending physicians decided the treatment scheme based on their experience in this reference centre and patient consent (Schubach et al. 2005, Vasconcellos et al. 2010).

We evaluated the following lesion characteristics: lesion area (< 700 mm^2^, > 700 mm^2^), evolution time (< 60 days, > 60 days), presence of secondary infection prior to antimonial treatment, and response to treatment using epithelialisation and total healing.

Lesion area was determined using the ellipse area formula, which is the typical shape of CL lesions: (transverse diameter/2) X (longitudinal diameter/2) X π. We measured lesion diameter at the external margins of the lesion border using a transparent ruler.

We defined secondary infection as the isolation of bacteria or fungi from a cultured lesion fragment. We also evaluated the following signs and symptoms suggestive of secondary infection: secretion, pain, burning sensation and pruritus.

We defined epithelialisation as a lesion that no longer presented with erosion and crusting. However, the lesion at this stage could present with desquamation, infiltration and/or erythema. We evaluated epithelialisation at medical appointments every 10 to 20 days from the start of treatment. Patients were followed up after epithelialisation at one, two, three, six, nine and 12 months to evaluate progression to total wound healing. We defined total healing or resolution of the lesion as an epithelialised lesion without desquamation and infiltration and with or without slight residual erythema.

We analysed the association of the occurrence of secondary infection with the categorical variables using Fisher’s exact test. We evaluated continuous variables (rejected by the Shapiro-Wilk normality test) using the Mann-Whitney non-parametric test. The exploratory survival analysis of epithelialisation and of total healing times, stratified by the occurrence of secondary infection and signs and symptoms suggestive of secondary infection, was performed using the Kaplan-Meier method. Patients who abandoned post-treatment follow-up were censored at their last visit. The log-rank test indicated significant survival curves at the 5% level.

Analyses were performed using R version 3.0 R Core Team software (2014) and the Statistical Package for the Social Sciences version 16 (SPSS, Inc., Chicago, IL, USA). We used a significance level of 5% in all statistical tests.

The INI/Fiocruz Research Ethics Committee approved this study under number CAAE 19704113.0.0000.5262.

## RESULTS

We included 25 patients with CL, aged nine to 76 years (median of 31 years), and 84% of the patients were men. Patients presented with one to five lesions, and 15 patients exhibited a single lesion. The number of lesions did not affect epithelialisation or total healing time. Thirteen patients presented lesions on the lower limbs, and seven patients presented lesions on the upper limbs. Four patients exhibited lesions on the trunk, and one patient presented with head and neck lesions.

All patients had a confirmed parasitological diagnosis. Amastigotes were visualised on culture in 20 (80%) patients, and histochemical or immunohistochemical analysis of lesion fragments identified amastigotes in 14 (56%) patients.

The median time of lesion evolution at the first visit was 60 days (range 15 to 330 days). Lesion area varied from 94 to 10,387 mm^2^ (median 942 mm^2^). Some signs and symptoms suggestive of secondary infection were observed in 80% of the patients (60% presented with secretion, 44% with pain, 44% with pruritus, and 40% with a burning sensation). Five patients exhibited co-morbidities, such as arterial hypertension, anaemia, chronic obstructive pulmonary disease, alcoholism, and diabetes mellitus.

Systemic and/or topical antibiotic were used prior to biopsy in 88% (n = 22) of the patients, and 12% (n = 3) used a systemic and/or topical antifungal agent. The most commonly used systemic antibiotic was cephalexin, followed by amoxicillin and sulfamethoxazole + trimethoprim alone or in combination with other systemic or topical antibiotics. Seven patients used topical neomycin + bacitracin, one patient used mupirocin, and one patient used rifampicin. Some patients could not specify the topical antibiotics used. Other systemic antibiotics included clindamycin, benzathine penicillin, ciprofloxacin, norfloxacin, and tetracycline. Antifungals included oral itraconazole, topical miconazole and topical ketoconazole.

Secondary infection of the lesion was found in 40% (n = 10) of the patients. The most frequently isolated bacterium was *S. aureus*. All of these patients exhibited at least one sign or symptom suggestive of secondary infection, and most of them had received previous antibiotic therapy. Most isolates of *S. aureus* were resistant to several antibiotics. Three patients did not use antibiotics or did not remember the substances used, and four patients used antibiotics that were not appropriate for the subsequently isolated bacteria. Only one of the isolated Gram-positive bacteria was sensitive to the previously used antibiotic. The isolated Gram-negative bacteria were not resistant to standard antibiotics. *Candida parapsilosis* was isolated in two patients (Table I). Identification of the parasite was performed in eight cases; *L. (V.) braziliensis* was the only identified species.

**TABLE I:** Isolated microorganism, antibiotic therapy in the 30 days previously to the skin biopsy, and signs and symptoms suggestive of secondary infection observed in ten patients with cutaneous leishmaniasis (CL) lesions in Rio de Janeiro between 2013-2016

Patient	Isolated microorganisms	Antibiotic therapy (30 previous days)	Signs and symptoms suggestive of secondary infection	Resistance to tested antibiotics
1	*Staphylococcus aureus*	NI	Pain, burning sensation and pruritus	CL, ER, GE, PE
2	*S. aureus*	NI	Pruritus	CL, ER, GE, PE
3	*Pseudomonas aeruginosa*	CL, CE	Secretion, pain and pruritus	NR
4	*S. aureus*	S+T, N+B	Secretion, pain and burning sensation	NR
5	*Enterococcus faecalis*	CE, N+B	Secretion and burning sensation	CI, RI, TE, LE, ST
6*	*P. aeruginosa*	AM	Secretion and burning sensation	NR
7	*Candida parapsilosis*	S+T, N+B	Secretion	Not A
8	*S. aureus*	AM	Secretion, burning sensation and pruritus	PE
9	*S. aureus*	NA	Burning sensation and pruritus	PE, TE
	*Streptococcus pyogenes*			ASTNR
10	*C. parapsilosis*	NA	Secretion, burning sensation and pruritus	Not A

*: patient used itraconazole. Other patients did not use antifungals for the 30 days before the biopsy. CL: clindamycin; CE: cephalexin; S + T: sulfamethoxazole + trimethoprim; N + B: neomycin + bacitracin; AM: amoxicillin; ER: erythromycin; GE: gentamycin; CI: ciprofloxacin; RI: rifampicin; TE: tetracycline; LE: levofloxacin; PE: penicillin; ST: streptomycin; NI: patient did not inform; NA: patient did not use antibiotic; NR: no resistance; ASTNR: antibiotic sensitivity test not recommended; Not A: not applicable.

The occurrence of secretion, a burning sensation and/or pruritus was more frequent when secondary infection was present, but only the burning sensation was statistically significant (Table II).

**TABLE II:** Analysis of categorical variables according to the occurrence of secondary infection in patients with cutaneous leishmaniasis (CL) treated in Rio de Janeiro between 2013-2016

Variables		Secondary infection		p-value

		Yes (N = 10)	No (N = 15)	
Gender	Men	8	13	1.000
	Women	2	2	
Age	≤ 18 years	2	2	1.000
	> 18 years	8	13	
Secretion	Yes	7	8	0.678
	No	3	7	
Pain	Yes	3	8	0.414
	No	7	7	
Burning sensation	Yes	7	3	0.034*
	No	3	12	
Pruritus	Yes	6	5	0.241
	No	4	10	
Number of lesions	One lesion	6	9	1.000
	≥ two lesions	4	6	
Area of the lesion	< 700 mm^2^	4	7	1.000
	> 700 mm^2^	6	8	
Time of evolution of the lesions	< 60 days	4	10	0.241
	> 60 days	6	5	
Co-morbidities	Yes	2	3	1.000
	No	8	12	
Lesion location	Lower limbs	6	7	0.688
	Other locations	4	8	

*: significant p-value.

All patients presented epithelialisation of the lesions. Time for epithelialisation varied between 9 and 124 days, with a median of 51 days. One patient, who abandoned follow-up prior to epithelialisation, returned to the medical appointment after one year with the lesion totally healed.

There was no significant difference in epithelialisation time between patients with and without secondary infection. However, lesions with secretion or a burning sensation exhibited longer epithelialisation time than lesions from patients without these characteristics (Table III).

**TABLE III:** Kaplan-Meier analysis of the time observed until epithelialisation of the lesions of 25 patients with cutaneous leishmaniasis (CL) treated in Rio de Janeiro between 2013-2016

Variable		n (%)	Time for epithelialisation (days)	Log rank p-value

			Median (Percentile 70;90)	
Secondary infection	presence	10 (40)	54 (71; 123)	0.395
	absence	15 (60)	49 (89; 117)	
Secretion	presence	15 (60)	63 (96; 121)	0.023*
	absence	10 (40)	28 (46; 98)	
Pain	presence	11 (44)	77 (96; 119)	0.522
	absence	14 (56)	39 (54; 121)	
Burning sensation	presence	10 (40)	77 (119; 124)	0.019*
	absence	15 (60)	42 (54; 96)	
Pruritus	presence	11 (44)	49 (79; 124)	0.975
	absence	14 (56)	55 (77; 119)	

*: significant p-value.

Twenty-one patients (72%) presented total healing of the lesions, and the time to achieve healing ranged from 26 to 301 days (median of 173 days). The remaining four patients discontinued follow-up. However, these patients were rescued after one year of treatment, when their lesions were totally healed.

There was no significant difference in the time required for total healing of the lesions of patients with secondary infection compared with patients without infection. None of the signs and symptoms indicative of secondary infection influenced the total healing time of the CL lesions (Table IV).

**TABLE IV:** Kaplan-Meier analysis up to the observed time for total healing of the lesions of 25 patients with cutaneous leishmaniasis (CL) attended in Rio de Janeiro between 2013-2016

Variable		n (%)	Time for total healing (days)	Log rank p-value

			Median (Percentile 70; 90)	
Secondary infection	presence	10 (40)	184 (231; 267)	0.292
	absence	15 (60)	161 (182; 231)	
Secretion	presence	15 (60)	173 (226; 231)	0.781
	absence	10 (40)	182 (231; 301)	
Pain	presence	11 (44)	182 (231; 233)	0.770
	absence	14 (56)	161 (226; 231)	
Burning sensation	presence	10 (40)	156 (196; 231)	0.304
	absence	15 (60)	182 (231; 233)	
Pruritus	presence	11 (44)	196 (231; 301)	0.449
	absence	14 (56)	173 (226; 231)	

## DISCUSSION

This study investigated the occurrence of secondary infection and the signs and symptoms suggestive of this infection and their influence on epithelialisation and total healing times of the lesions of CL patients. Most of the study population were young male adults with a single cutaneous lesion located primarily in the lower limbs, which is consistent with previous studies (Schubach et al. 2005).

CL is a disease that depends on exposure to the sand fly vector, which is fundamentally a forest inhabitant. However, the sand flies adapted to the environment around human habitation in endemic areas of ancient colonisation in Brazil, such as the state of RJ, and may infect any age in these areas. This exposure explains why the patients of different age groups are affected, but with greater involvement of adults, who are also exposed during outdoor activities. The predominance in the male gender reflects the greater exposure of men to the bite of the vectors in certain professional activities, such as soldiers training in the jungle (MS 2017).

The occurrence of secondary infection in lesions of the studied patients was similar to previous studies (Vera et al. 2001, Gonçalves et al. 2009, Sadeghian et al. 2011, Doudi et al. 2012). Patients with ulcerated CL lesions are subject to secondary infection. Salgado et al. (2016) suggested that the inflammation promoted by the presence of *Leishmania* parasites and triggered by the innate immune system creates an environment that is suitable for the development of some commensal bacteria but hinders the development of less adapted parasites.

Chronic wound infection hampers the healing process, particularly when biofilms are formed, because biofilms reduce germ elimination by antimicrobials or the innate and adaptive host immune response (Høiby et al. 2015, Vyas & Wong 2016). However, this study did not analyse the biofilms in CL-specific lesions. The use of non-specific treatments, such as physical wound debridement that would destroy biofilms without topical destruction of parasites, are not an ethical choice in the New World because of the non-negligible risk of mucosal leishmaniasis. The first choice for specific treatment in the CL of the New World is pentavalent antimonial treatment, but the dose and route of administration must be evaluated according to the risk-benefit for each patient (WHO 2010).

Two bacteria were isolated in the same lesion in only one studied patient. Fontes et al. (2005) also observed contaminations of cutaneous lesions by a single microorganism in most cases. The competitive environment between the most adaptable bacteria may explain the small diversity of isolated microorganisms (Salgado et al. 2016). Another explanation for the absence of microorganism diversity in the studied lesions is the method used to collect the sample, with a thorough asepsis of the lesion prior to the collection of the fragment through a biopsy procedure. We aimed to reduce the isolation of microorganisms that were only colonising the superficial layer of the lesions. Sample collection was performed using a swab after cleaning with saline in previous studies, and a greater diversity of microorganisms was observed (Gonçalves et al. 2009, Salgado et al. 2016).

The negativity of the cultures and the low diversity of the isolated bacteria in the cutaneous lesions in this study may also be related to the previous use of antibiotic therapy (Navarro et al. 2009). The use of previous antibiotic therapy may have influenced the isolation of bacteria (Fontes et al. 2005, Gonçalvez et al. 2009, Yeboah-Manu et al. 2013). However, we cannot conclude that the previously used antibiotics were effective or used properly because this use occurred prior to the medical care at INI. Data on previous antibiotic therapy were obtained from patient reports, and it was not possible to determine the dose or duration of use.

*S. aureus* was the most frequently isolated bacterium, and it was also the most prevalent bacterium in other studies that evaluated the microbiota of cutaneous CL lesions (Fontes et al. 2005, Doudi et al. 2012, Salgado et al. 2016). *S. aureus* is found in normal skin microbiota, and ulceration may be a gateway to infection development. This bacterium also produces enzymes that lyse tissue components, which facilitates invasion (Doudi et al. 2012).

*P. aeruginosa* and *Enterococcus faecalis* are commensal microorganisms that act as opportunistic pathogens (Brouwer et al. 2016). *P. aeruginosa* infection is generally confined to the skin or its appendages in immunocompetent patients. *E. faecalis* is part of the amphibiotic microbiota of the gastrointestinal tract. Contamination may occur from person to person or through the consumption of contaminated food or water (Facklam et al. 1999). *Streptococcus pyogenes* is often associated with primary infections of the pharynx and tonsils, and its transmission from person to person occurs via direct contact or secretions (e.g., sneezing and coughing) (Brouwer et al. 2016). All of these bacteria were isolated from CL lesions in previous studies (Shirazi et al. 2007, Gonçalves et al. 2009).

*Candida* sp was also isolated from CL lesions in this study in a lower proportion, similar to Fontes et al. (2005). The isolation of this fungus, which is part of the human microbiota, is a reflection of its opportunistic nature (Kashem & Kaplan 2016).

One limitation of the present study was that the biopsies were performed at the border of the lesions for the parasitological confirmation of CL. A biopsy sample from the base of the debrided ulcer is recommended by the European Society of Clinical Microbiology and Infectious Diseases (ESCMID) guidelines when moderate to severe soft tissue infection is suspected in chronic wounds (Høiby et al. 2015). However, this sampling is not a routine procedure for CL management in Brazil. The use of cleansing methods at the biopsy sites may also influence the results of microbiological studies (Schwarzkopf & Dissemond 2015). Another weakness of the study was the small number of allocated patients, which reflects the decrease in CL cases in Brazil in recent years. The number of patients with secondary infection made it difficult to verify the influence of each microorganism on epithelialisation and total healing times using survival analyses.

The presence of secondary infection did not influence epithelialisation or the total healing time of the lesions. However, most patients with secondary infection presented with secretion and reported a burning sensation, both of which are associated with a longer epithelialisation time. The presence of purulent secretion suggests infection in ulcerated lesions (Fierheller & Sibbald 2010). Large and purulent lesions generally exhibit higher positivity in fungal or bacterial cultures (Fontes et al. 2005). “Burning sensation” is primarily due to chronic inflammation in reaction to biofilms. Consequently, this categorical variable was represented significantly more often when microorganisms were isolated in CL ulcers. These two indirect clinical parameters of wound infection (secretion and burning sensation) were better indicators of wound healing impairment than microorganism isolation, pain and pruritus. The clinical wound infection markers “pain” and “pruritus” are likely to be modified by altered nociceptive signals during the course of CL (Borghi et al. 2017).

Our borderline proof of clinically infected CL lesions inhibiting wound healing supports infection prevention using wound hygiene and antisepsis with only narrow-spectrum systemic and topic antibiosis in the treatment of ulcerated CL lesions, which is consistent with the antimicrobial stewardship (Lipsky et al. 2016) recently advocated for all chronic wounds (O’Donnell & Guarascio 2017).
